# A Novel Association Between Loop Diuretic Therapy and Repigmentation in Two Patients With Vitiligo

**DOI:** 10.7759/cureus.16836

**Published:** 2021-08-02

**Authors:** Mary Arndt, Rachel Sennett, Amanda Marsch

**Affiliations:** 1 Department of Medicine, Lake Erie College of Osteopathic Medicine, Bradenton, USA; 2 Department of Dermatology, University of California San Diego, La Jolla, CA, USA

**Keywords:** vitiligo, repigmentation, loop diuretics, furosemide, photosensitivity, photosensitizing, drug-induced pigmentation

## Abstract

Two patients with longstanding vitiligo presented with repigmentation in sun-exposed areas as a previously unreported phenomenon coinciding with initiating loop diuretic therapy. Loop diuretics antagonize Na-K-Cl cotransporters and have been associated with a variety of cutaneous adverse effects, such as bullous pemphigoid and photosensitivity, but have yet to be cited as drugs associated with vitiligo repigmentation. This report explores the direct and indirect influence loop diuretics may have on inducing pigmentation changes.

## Introduction

Vitiligo is an autoimmune disease with a pathogenesis encompassing genetic, environmental, and metabolic factors. The innate and adaptive immune systems are implicated in melanocyte destruction, which presents clinically as patches of depigmentation [1]. Mainstay treatment options for repigmentation include phototherapy, topical and systemic immunosuppressants, and surgical grafting techniques, which are all thought to stimulate activation of melanoblasts in the hair follicle and/or intact marginal epidermal melanocytes which subsequently repopulate the affected interfollicular epidermis [1,2]. However, the mechanisms through which these treatment modalities promote repigmentation remain elusive.

Loop diuretics (LD) antagonize Na-K-Cl cotransporters (NKCC) and are conventionally prescribed as diuretics to treat hypertension and edema. Certain LD are classified as sulfonamides, which are known photosensitizers that can increase susceptibility to cutaneous photoallergic and phototoxic reactions in patients taking these medications. In this report, we review two cases of patients with longstanding vitiligo who both presented with repigmentation in sun-exposed areas after initiation or adjustment in their loop diuretic therapy. The mechanisms through which systemic LD therapy may contribute to pigmentary changes, particularly in patients with longstanding depigmentation in the context of vitiligo, merits further inquiry.

## Case presentation

**Figure 1:**
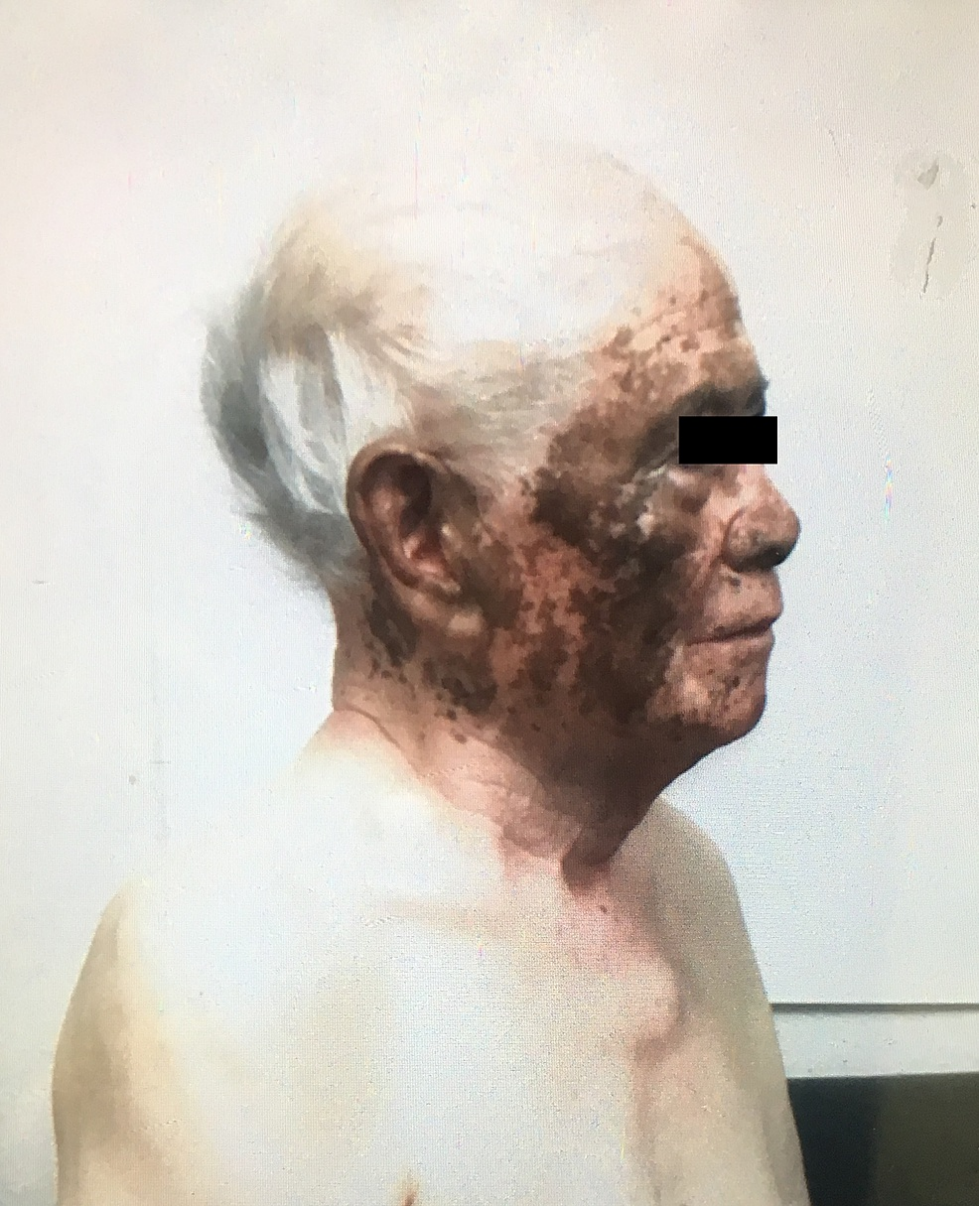
Patient 1 with scattered brown patches of pigment coalescing in sun exposed areas after initiating loop diuretic therapy.

Patient 1: An 88-year-old man with a history of end-stage renal disease and long-standing generalized vitiligo initially presented to the dermatology clinic with concerns of several years of repigmentation in sun-exposed areas which began upon initiating furosemide three years prior to presentation. Spotty pigmentation remained despite discontinuation of furosemide for eight months with the start of his dialysis. Pictures of the patient at time of furosemide discontinuation demonstrated brown macules and patches of pigment over his face, extensor wrists, and dorsal hands (Figure 1).

**Figure 2:**
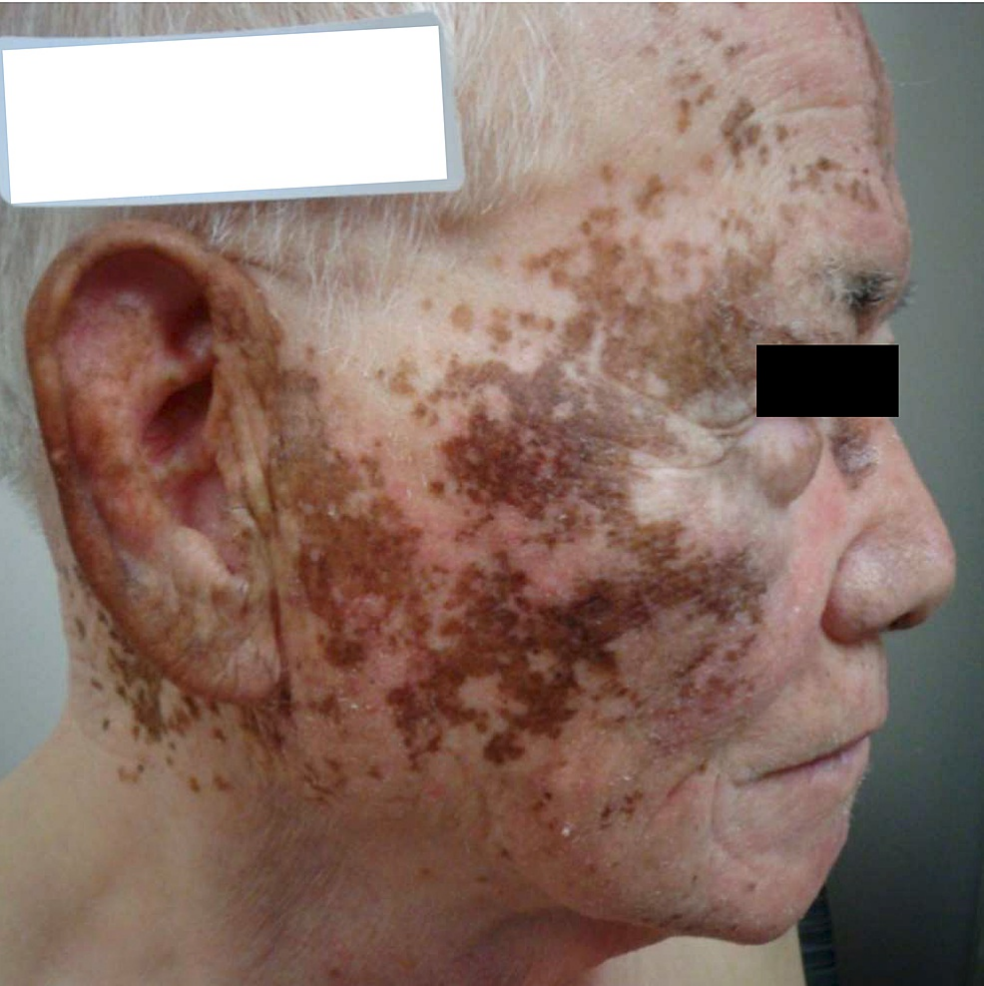
Four months after staring MBEH, Patient 1 displays scattered brown macules and geographic patches of pigment on face and ears, faded since initial visit. MBEH (monobenzyl ether of hydroquinone)

**Figure 3:**
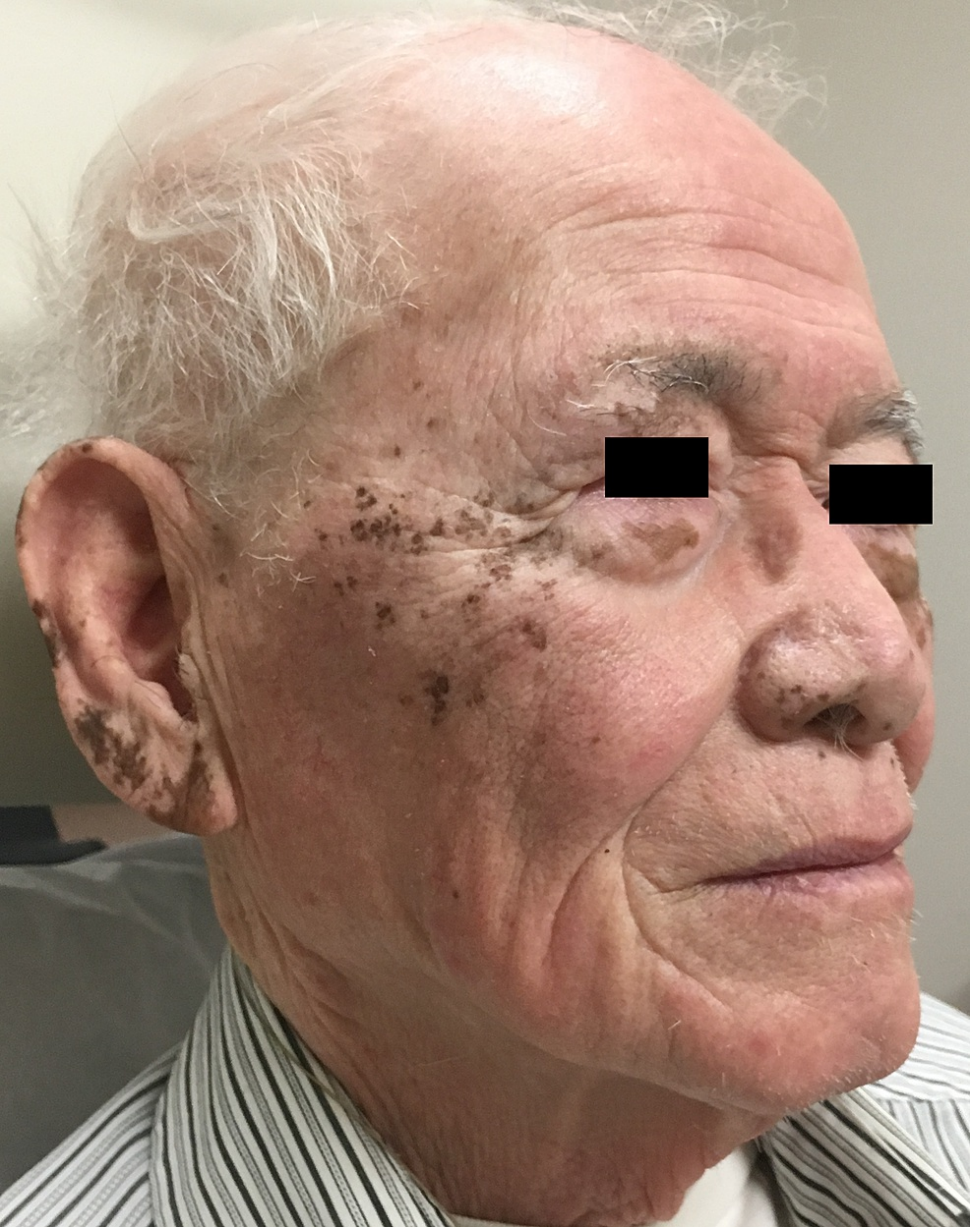
1.5 years after starting MBEH, Patient 1 displays only a few persistent pigmented macules over bilateral ears and cheeks. MBEH (monobenzyl ether of hydroquinone)

Of note, he had switched to ethacrynic acid from furosemide in the past year, prior to presentation, during which time the pigmentation remained stable. Physical exam revealed persistent scattered brown macules and geographic patches of pigment over his ears, cheeks and nose, along with similar involvement of the dorsal hands. As the patient was motivated to pursue depigmentation, topical monobenzyl ether of hydroquinone (MBEH) was initiated. At follow-up four months later, the patient presented with notably faded brown macules and geographic patches on his face and ears (Figure 2) with continued pigmentary clearance at subsequent follow-up after another year (Figure 3).

Patient 2: A 45-year-old man with heart failure and an eight-year history of generalized vitiligo similarly presented to the dermatology clinic noting recent repigmentation in sun-exposed areas which he believed had started with initiation of torsemide six months prior. His medications also included chlorthalidone, which had been initiated in the weeks just prior to presentation. Notable prior medications also included over two years of bumetanide prior to switching to torsemide. Physical exam revealed brown geographic patches on his cheeks, with brown macules coalescing into smaller patches on his forehead, temples, ears, hands, and distal forearms. Patches had an accentuated, hyperpigmented rim but lacked associated erythema. At the time of presentation, the patient opted to initiate light therapy for repigmentation, but unfortunately passed away shortly afterward from complications secondary to heart failure. Photo consent was unable to be obtained.

## Discussion

Sulfonamide medications, which include loop diuretics (LD) furosemide, torsemide, and bumetanide, are known photosensitizers that can lead to drug-induced phototoxic reactions and the potential for delayed hyperpigmentation in susceptible patients. Melanogenesis is stimulated by inflammation brought on when phototoxic agents absorb light and form free radicals [3]. This concept is purposefully employed for repigmentation in vitiligo patients treated with phototherapy together with systemic psoralen, a potent photosensitizer [1,3]. The observation that both patients described here experienced photodistributed dyschromia after initiating or switching to a new sulfonamide diuretic suggests that drug-induced photosensitization could similarly underlie their seemingly spontaneous repigmentation - particularly in Patient 1 who had experienced complete and stable pigment absence for decades prior to his presentation.

Drug-induced pigmentation is a separate and distinct entity. Four recognized mechanisms of drug-induced pigmentation include accumulation of melanin, accumulation of the drug itself, drug-induced induction of new pigment synthesis, and deposition of heme iron secondary to drug-induced vascular damage [4]. Drug-induced melanin accumulation is exacerbated by sun exposure which in and of itself stimulates melanogenesis. The process is otherwise triggered directly by the drug, a drug-induced inflammatory response or by the drug forming a complex with melanin which can deposit in dermal macrophages and impair melanin clearance.

Separately, LD have an indirect role in stimulating prostaglandin production, which has immunomodulatory effects and the potential to stimulate melanocyte proliferation [1,5]. Perhaps an increase of prostaglandins contributed to LD-associated vitiligo repigmentation in these two cases.

Finally, in addition to a role for perifollicular melanoblasts and perilesional marginal melanocytes in promoting repigmentation, recent studies suggest eccrine sweat glands might also harbor melanocyte precursors capable of contributing to pigment restoration in patients with vitiligo [2,6,7]. Secretory cells in the eccrine coil have also been found to express the NKCC1 cotransporter with observed bumetanide-sensitive NaCl secretion [2,8]. The initiation of a new LD could potentially directly affect eccrine secretory cell activity, with downstream implications for the melanoblasts sheltered nearby within the eccrine coil. Recent literature describes an expanding role for NKCC1 dysregulation itself in the pathophysiology of varying disease states, and inflammation-induced reactive oxygen species have been implicated in activating and upregulating NKCC1 in inflammatory bowel disease and ischemic stroke [2,6,9]. If NKCC1 dysregulation plays any role in vitiligo pathogenesis, systemic LD therapy might affect pigmentation through a completely novel mechanism.

Literature review revealed one case report associating furosemide therapy with the onset of progressive pigmented macules [10]. No reports, however, associate LD therapy with repigmentation in patients with vitiligo.

## Conclusions

The relationship between the initiation of LD therapy and the development of vitiligo repigmentation in these two patients suggests a drug induced phenomenon, although we cannot exclude spontaneous repigmentation or an additional drug or mechanism contributing to the reported findings. Nevertheless, potential mechanisms for vitiligo skin repigmentation in association with LD warrant further exploration including drug induced photosensitization, pigmentation, prostaglandin immunomodulatory effects and eccrine sweat gland NKCC1 regulation.
